# Mixed-method evaluation of interactive provider dashboards for comparison of outpatient antibiotic prescribing for respiratory and otic conditions in walk-in clinics

**DOI:** 10.1017/ash.2026.10365

**Published:** 2026-05-11

**Authors:** Kelly M. Percival, Kimberly C. Dukes, Gosia S. Clore, Stacey Hockett Sherlock, Dilek Ince, Nathan Shaw, Patrick M. Kinn, Lukasz Weiner, Christina Kopp, Mary Vaughan Sarrazin, Daniel J. Livorsi

**Affiliations:** 1 Department of Pharmaceutical Care, University of Iowa Health Care, Iowa City , IA, USA; 2 Department of Internal Medicine, The University of Iowa Roy J and Lucille A Carver College of Medicine, Iowa City, IA, USA; 3 Center for Access and Delivery Research and Evaluation (CADRE) Iowa City (VRHRC-IC), Iowa City Veterans Affairs (VA) Healthcare System, Iowa City, IA, USA; 4 Department of Family Medicine, The University of Iowa Roy J and Lucille A Carver College of Medicine, Iowa City, IA, USA; 5 Department of Pediatrics, Division of Infectious Diseases, The University of Iowa Roy J and Lucille A Carver College of Medicine, Iowa City, IA, USA

## Abstract

**Objective:**

We evaluated how antibiotic use changed after implementation of a multifaceted intervention that sent providers individualized peer-comparison feedback on their antibiotic use for respiratory conditions that do not warrant antibiotics (never-events).

**Design:**

An interrupted time-series analysis was performed with a baseline (January 2018–January 2020) and intervention period (November 2021–December 2023), while controlling for COVID-19 era (February 2020–February 2022).

**Setting:**

Walk-in ambulatory clinics.

**Participants:**

Providers caring for patients in walk-in clinics.

**Methods:**

We conducted a mixed-methods study across 7 walk-in clinics in one health system. We included data from visits from 2018–2023 and conducted 17 semi-structured interviews with 10 providers.

**Results:**

After intervention implementation, antibiotic use for all visits decreased 8% (RR 0.92, 95% CI 0.86–0.97), then began to increase by 1% per month (RR 1.01, 95% CI 1.00–1.01). Once the intervention started, the use of never-event diagnostic codes decreased by 24% (RR 0.69–0.83) and continued to decrease by 1% per month (RR 0.99, 95% CI 0.98–0.99). Antibiotic use for never-event visits showed no immediate change after the intervention started (RR 0.80, 95% CI 0.61–1.04), then decreased by 3% per month (RR 0.97, 95% CI 0.96–0.98). Some providers valued receiving feedback on the metric; others admitted to shifting their codes.

**Conclusions:**

Delivering feedback to walk-in clinic providers was associated with temporary reductions in antibiotic-prescribing across all visits but also changes in diagnostic coding (ie, “gaming”). Antibiotic stewardship programs should monitor for changes in both when implementing new outpatient metrics.

## Introduction

Antibiotic overuse is common across all areas of health care, including in urgent care (UC) and other walk-in clinics, especially for respiratory viral infections, such as bronchitis and nonspecific upper respiratory infection, that should never require antibiotic therapy.^1–3^ About 50% of antibiotic use for acute respiratory conditions is unnecessary.^1^ The overuse of antibiotics drives antibiotic resistance and other antibiotic-related adverse effects.^4^

The CDC Core Elements for Outpatient Antibiotic Stewardship highlights the importance of antibiotic stewardship in all ambulatory settings, including walk-in clinics.^5^ According to the CDC, a high-priority target for intervention is antibiotic prescribing for respiratory conditions where an antibiotic is not indicated. Reducing overprescribing by 33% (OR 0.67, 95% CI 0.54–0.82) has been seen for these conditions in walk-in clinics and Emergency Departments (EDs) with multifaceted interventions, which have typically included an audit-and-feedback component with comparison to peers along with education to providers and patients and physician champions.^6^ However, only a few of these prior reports evaluated whether this approach to audit-and-feedback motivated providers to change their diagnostic coding.^7,8^ This phenomenon is known as diagnostic shifting, which refers to deliberately avoiding surveillance by purposely choosing diagnostic codes not included in the metric.^7^

The goal of the project was to evaluate implementation of a multifaceted stewardship intervention across walk-in clinics, including staff attitudes, changes in antibiotic-prescribing and in the use of diagnostic codes. The intervention primarily consisted of sending providers individualized peer comparison feedback reports on their antibiotic use for respiratory and otic conditions where antibiotics are not recommended.^9^ This was named the “never antibiotic event prescription rate” or “never-events metric” for short.

We hypothesized that, after implementation of our intervention, antibiotic-prescribing would decrease across all visits and specifically for never-event visits. We also hypothesized that providers would start using never-event codes less frequently once the intervention began.

## Methods

We performed an interrupted time-series analysis to assess changes in antibiotic use and diagnostic coding in a before-and-after, quasi-experimental mixed-methods study across 7 walk-in clinics. We defined a baseline period (January 2018–January 2020), a COVID-19 period (February 2020–February 2022), and an intervention period (November 2021–December 2023). The COVID-19 period was roughly defined by the onset of the COVID pandemic in the United States to the CDC’s lifting of most indoor masking recommendations.

The University of Iowa Institutional Review Board approved all aspects of the study.

### Clinical context

University of Iowa Health Care has three UC clinics and four QuickCare (QC) clinics with ∼11,000 visits per month across all seven clinics. Urgent Care clinics provide walk-in visits with on-site x-ray, laboratory testing, IV therapy, and procedural capabilities including laceration repair and fracture immobilization. QC clinics provide walk-in care for minor acute illnesses and injuries with basic point-of-care testing. These clinics most commonly treat acute respiratory infections, otitis media, urinary tract infections, gastrointestinal, musculoskeletal, and skin related conditions. These clinics are primarily staffed by nurse practitioners (NPs) and physician assistants (PAs). The health system has utilized a web and mobile based app for antibiotic stewardship (AS) information since 2016; this app has institutional empiric treatment guidelines for all common infectious conditions, including pneumonia, bronchitis, otitis media and other acute respiratory infections, for inpatient and outpatient treatment. Prior to the intervention, no AS initiatives or resources were in place in these clinics, because the health system has no dedicated resources for outpatient AS. This project was started to further justify the need for AS resources in the outpatient setting.

### Intervention

Our intervention primarily used the following implementation strategies to improve antibiotic-prescribing for acute-respiratory conditions: (1) audit-and-feedback to providers with comparisons to their peers, (2) education, and (3) clinician prompts (ie, order sets).

### Provider feedback with peer-comparison: metric development and delivery

The antibiotic stewardship team collaborated with the leadership of the UC/QC clinics to provide data and feedback regarding antibiotic usage for walk-in clinic providers. The determination of respiratory and otic conditions which never require an antibiotic was done using ICD-10 codes (Supplement 2) recorded on antibiotic prescription records and encounters based on previously published classifications.^9^ The never antibiotic event prescription rate was calculated for each individual provider by taking the number of antibiotics prescribed for a never-event visit divided by the number of unique encounters for a never-event. This was done for all providers regardless of patient volumes. All ICD-10s associated with the encounter and antibiotics that could be used for a respiratory tract infection were evaluated; if any diagnostic codes were for a condition that may require an antibiotic, then that visit was not included in the never-event metric.

To provide feedback on this metric, an information technology specialist (0.5 FTE) assigned to the stewardship team created a visual dashboard of provider data using Tableau® software (Supplementary figures 1,2,3). The dashboard data for supplementary figures 1 and 3 were unique to the individual provider and were updated quarterly.

The dashboard was distributed to the providers via a universal email with a link to the dashboards that were unique to the individual provider based on their Tableau® login. The dashboard could be accessed at any time by providers with the link. The e-mail was composed by the antibiotic stewardship team; it took no more than 15 minutes per quarter to prepare and was sent by the UC/QC NP/PA supervisor. (Supplementary figure 4) The email was the same each quarter for all providers with no summary statements to indicate their personal performance. The quarterly dashboard was first distributed in November 2021 with data from July–October 2021 and continues to be distributed at the end of every fiscal year quarter. In addition to this dashboard, the provider’s individual monthly prescribing rate for never-events is included in a monthly quality report card sent to the provider with multiple other metrics that are distributed by UC/QC leadership. Other metrics included are patients seen per hour, average patient time spent in clinic, billing time, meeting attendance, how often providers did not take lunch, and how often providers had unplanned absences or stayed beyond their scheduled shift.

### Education

The same education session was delivered by an AS pharmacist in two separate meetings to providers who primarily worked in Urgent Care and in QuickCare Clinics in September 2021 prior to the never-event metric data being shared. Due to limited resources, further education sessions were not conducted. For any provider who joined the clinics after September 2021, the metric was introduced by the ARNP supervisor. Around 35% of providers who saw patients during the intervention period attended the initial education. The initial education session included an overview of the rising rates of antibiotic resistance along with the goals and the importance of (AS) in the outpatient setting. Then national data regarding the high and often unnecessary usage of antibiotics for respiratory tract infections was shown. Next, the institution-specific initiative of the never-metric was introduced with a detailed description of how the data was being obtained and utilized to create the prescription rate; dashboard images were shown and explained. The session concluded with review of the new order sets and questions.

### Order sets

All walk-in clinics use a common conditions order set to help promote guideline adherent treatment and streamline workflow. To support this stewardship initiative, two new conditions were added to the order set: bronchitis and upper respiratory tract infections. For both conditions, the order sets recommended against the use of antibiotics, but instead provided some potential supportive care treatments and had links to patient education. Order sets were used <10 times per month throughout the study period.

### Outcomes

To assess the effect of the intervention, we created a visit-level data set that allowed multiple visits per patient and per provider. Visits were excluded if care was only delivered via telehealth or if the patient was seen in the Emergency Department or hospitalized within 24 hours of their walk-in clinic visit. Telehealth visits were excluded due to the use of this visit type almost exclusively for patients with signs and symptoms consistent with COVID-19; as a result, these visits were felt to be inherently different than in-person visits.

The primary outcome was prescription of an antibiotic within 24 hours of walk-in clinic visit; only antibiotics that could be used for a respiratory tract infection were captured (Supplementary table 1). Examples of antibiotics that did not qualify include fidaxomicin, fosfomycin, nitrofurantoin and vancomycin. Secondary outcomes included (a) the frequency that a visit was associated with a qualifying never-event diagnostic code, and (b) the frequency that a qualifying antibiotic was prescribed for a never-event visit.

### Data analysis

To evaluate the impact of the intervention on antibiotic prescribing (or Never-Event coding), we used a Generalized Linear Mixed Model with a Poisson distribution and a log link function. This approach is useful for analyzing panel data (ie, longitudinal data) as it handles both cross-sectional variation and longitudinal changes, allowing us to examine variation across physicians and over time.^10^ By incorporating a random intercept for each provider, the model accounts for longitudinal nesting and controls for unobserved heterogeneity in individual clinical practice. The random intercept also provides a mechanism for partial pooling, by which physician-level trajectories are “shrunk” or pulled toward the average population trend. This technique helps to stabilize estimates, particularly for subjects or groups with limited data points or extreme values. We included the COVID-19 period (February 2020–February 2022) as a fixed-effect to account for the changes in epidemiology and care delivery that happened during that time. Other covariates included in the model were clinic location, season, patient’s age, race, and total comorbidities. SAS Enterprise Guide Version 8.3 Update 7 (8.3.7.202) was used to analyze the data.

### Qualitative data collection and analysis

To assess the acceptability and perceptions of the intervention, trained qualitative researchers (KD, SHS) conducted two rounds of semi-structured in-depth interviews (a set of open-ended questions and opportunities for interviewees to identify new topics; averaging ∼30 mins) with ARNP and PA-C providers in 7 clinics in January–March 2023 and June–July 2023, recording them with interviewee consent, then transcribing audio recordings. We imported transcripts into qualitative data management software MAXQDA (VERBI software). We developed a codebook using deductive and inductive codes and then conducted consensus-based thematic analysis.^11,12^ Qualitative methods were described previously.^13^

## Results

### Quantitative results

After excluding 54,060 visits seen through telehealth and 13,494 visits that were followed by an ED or hospital admission, there were 445,349 visits, performed by 143 providers, included for 159,471 unique patients. Median age was 27 (IQR 18–44) and 61% were female (Table 1). There were 278,704 (62.6%) visits seen by an NP, 152,280 (34.2%) by a PA, and 14,365 (3.2%) by a physician. Never-events accounted for 44,416 (10.0%) of all visits. Antibiotics prescribed are described in supplementary table 2.

**Table 1.** Patient and clinic visit characteristics

		Pre-intervention			Post-intervention	
Characteristics	All visits (%)	Visits with antibiotics (%)		Visits without antibiotics (%)	Visits with antibiotics (%)	Visits without antibiotics (%)
**Visits (n)**	445,349	50,421		167,964	53,761	173,203
**Age group, y**						
<18	102,114 (22.9)	12,673 (25.1)		33,272 (19.8)	16,836 (31.3)	39,333 (22.7)
≥18	343,235 (77.1)	37,748 (74.9)		134,692 (80.2)	36,925 (68.7)	133,870 (77.3)
**Gender**						
Female	271,609 (61.0)	31,459 (62.4)		102,503 (61.0)	32,731 (60.9)	104,916 (60.6)
**Race**						
White	335,782 (75.4)	40,537 (80.4)		125,669 (74.8)	42,908 (79.8)	126,668 (73.1)
African American/Black	41,906 (9.4)	3,427 (6.8)		16,329 (9.7)	3,753 (7.0)	18,397 (10.6)
Other/unknown	67,661 (15.2)	6,457 (12.8)		25,966 (15.5)	7,100 (13.2)	28,178 (16.3)
Comorbidities*						
Median (IQR)	0 (0–1)		0 (0–1)	0 (0–1)	0 (0–1)	0 (0–1)
**Provider type**						
Nurse practitioner	278,704 (62.6)	36,260 (71.9)		112,071 (66.7)	34,369 (63.9)	96,004 (55.4)
Physician assistant	152,280 (34.2)	13,104 (26.0)		50,597 (30.1)	17,944 (33.4)	70,635 (40.8)
Physician	14,365 (3.2)	1,057 (2.1)		5,296 (3.2)	1,448 (2.7)	6,564 (3.8)
**Never-event diagnostic codes**						
All never-events	44,416 (10.0)	2,621 (5.2)		23,064 (13.7)	1,663 (3.1)	17,068 (9.2)
Ear	14,743 (3.3)	1,106 (2.2)		7,068 (4.2)	990 (1.8)	5579 (3.2)
Respiratory	29,673 (6.7)	1,515 (3.0)		15,996 (9.5)	673 (1.3)	11,489 (6.6)
**All diagnostic codes**						
Bronchitis/bronchiolitis	5,130 (1.2)	619 (1.2)		2,763 (1.7)	244 (0.5)	1,504 (0.9)
COVID	9,091 (2.0)	10 (0.0)		2,054 (1.2)	51 (0.1)	6,976 (4.0)
Fever	7,884 (1.8)	164 (0.3)		3,483 (2.1)	161 (0.3)	4,076 (2.4)
Influenza	2,003 (0.4)	10 (0.0)		1,486 (0.9)	4 (0.0)	503 (0.3)
Pharyngitis	72,910 (16.4)	7,867 (15.6)		23,585 (14.0)	10,808 (20.1)	30,650 (17.7)
Pneumonia	2,809 (0.6)	726 (1.4)		392 (0.2)	1,359 (2.5)	332 (0.2)
Sinusitis	17,522 (3.9)	8,303 (16.5)		1,233 (0.7)	7,142 (13.3)	844 (0.5)
Suppurative otitis media	22,074 (5.0)	9,376 (18.6)		976 (0.6)	10,776 (20.0)	946 (0.5)
Non-suppurative otitis media	5,189 (1.2)	792 (1.6)		1,984 (1.2)	795 (1.5)	1,618 (0.9)
Other respiratory conditions	10,143 (2.3)	266 (0.5)		4,655 (2.8)	190 (0.4)	5,032 (2.9)
Viral upper respiratory infection	38,795 (8.7)	1,731 (3.4)		17,344 (10.3)	912 (1.7)	18,808 (10.9)

* Comorbidities included congestive heart failure, COPD, diabetes, chronic kidney disease, liver disease, malignancy, dementia, rheumatic disease, paralysis, neurological disease.

After implementation of the intervention, the frequency of antibiotic-prescribing for all visits changed from 23.1% to 23.7% (*P* < .001), the use of never-event codes changed from 11.8% to 8.3% (*P* < .001), and the use of antibiotics for never-event visits changed from 10.2% (2,621/25,685) to 8.9% (1,663/18,731) (*P* < .001).

**Figure 1.**
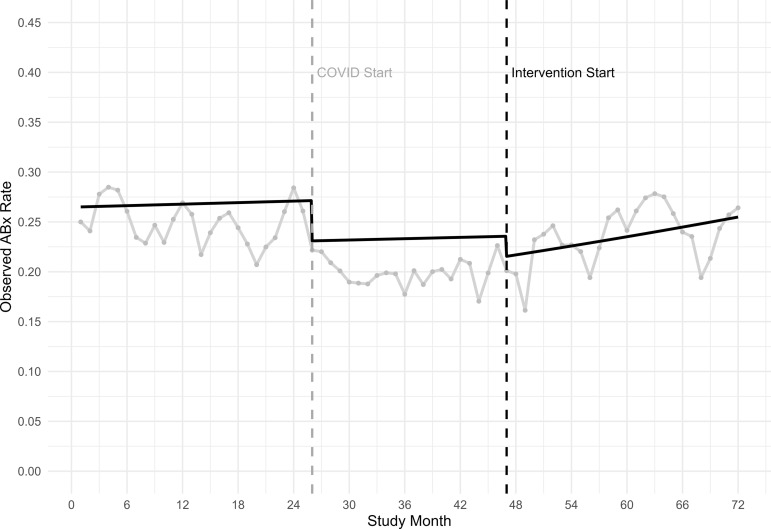
Model-adjusted trends in antibiotic-prescribing frequency overlaid on observed prescribing rates across 7 walk-in clinics, 2018–2023. Legend: Monthly observed antibiotic prescribing rate (ABx rate) for an antibiotic that could be used for a respiratory infection, from walk-in clinics during baseline months 1–25 (January 2018–January 2020), COVID-19 starts month 26 (February 2020), Intervention months 47–72 (November 2021–December 2023).

In the interrupted time-series analysis (Table 2), antibiotic use for all visits decreased 8% after the intervention started (RR 0.92, 95% CI 0.86–0.97; *P* < .004), then began to increase by 1% per month for the rest of the intervention period (RR 1.01, 95% CI 1.00–1.01; *P* < .001) (Figure 1). After the intervention started, the use of never-event diagnostic codes decreased by 24% (OR 0.69–0.83; *P* < .001) and continued to decrease by 1% per month (RR 0.99, 95% CI 0.98–0.99; *P* < .001) (Supplementary figure 5). Antibiotic use for never-event visits showed no immediate change after the intervention started (RR 0.80, 95% CI 0.61–1.04; *P* = .10), then decreased by 3% per month during the rest of the intervention period (RR 0.97, 95% CI 0.96–0.98; *P* < .001) (Supplementary figure 6).

**Table 2.** Primary and secondary outcomes for provider feedback with peer-comparison intervention across 7 walk-in clinics, 2018–2023

Outcome	Rate ratios (95% CI) relative to the baseline period^1^	*P*-value
**Frequency of antibiotic use for any visit, regardless of the diagnosis (primary outcome)**		
COVID era: immediate change from baseline^2^	0.85 (0.78–0.93)	<.001
COVID era: change in slope after COVID started relative to the baseline trend	1.00 (1.00–1.00)	.81
Intervention: immediate change from baseline^3^	0.92 (0.86–0.97)	.004
Intervention: change in slope after the intervention relative to the baseline trend	1.01 (1.00–1.01)	<.001
**Frequency of using never-event codes for visits** ^4^ **(secondary outcome)**		
COVID era: immediate change from baseline^2^	0.59 (0.51–0.68)	<.001
COVID era: change in slope after COVID started relative to the baseline trend	1.00 (1.00–1.00)	.18
Intervention: immediate change from baseline^3^	0.76 (0.69–0.83)	<.001
Intervention: change in slope after the intervention relative to the baseline trend	0.99 (0.98–0.99)	<.001
**Frequency of prescribing antibiotics for visits coded as never-events** ^4^ **(secondary outcome)**		
COVID era: immediate change from baseline^2^	1.17 (0.77–1.78)	.46
COVID era: change in slope after COVID started relative to the baseline trend	0.99 (0.98–1.00)	.09
Intervention: immediate change from baseline^3^	0.80 (0.61–1.04)	.10
Intervention: change in slope after the intervention relative to the baseline trend	0.97 (0.96–0.98)	<.001

1 The baseline period extended from January 2018–January 2020.

2 The COVID-19 era was defined as February 2020–February 2022.

3 The intervention period started in November 2021 and ended in December 2023.

4 Never-event codes included 555 International Classification of Disease (ICD)-10 diagnostic codes for respiratory or otic conditions that do not benefit from antibiotic therapy.

### Qualitative results

Ten providers (NPs and PAs) participated in 17 interviews. Table 3 provides additional exemplar quotes for themes discussed below.

**Table 3.** Exemplar quotes regarding the metric

**Metric may be prone to ‘gaming’ with clinicians making changes to their ICD-10 coding**	“… basically, they were saying, ‘These diagnoses we’re going to flag as ‘no,’ and these diagnoses we’re going to flag as ‘yes,’’ and I think everybody just changed what they were calling stuff to give antibiotics.” (Provider #2) “I just know that if I’m going to prescribe something for sinusitis and I don’t really think they need antibiotics, I’m probably going to call it ‘sinusitis” versus a URI.” (Provider #4)
**Difficulty of interpreting metric based on concerns about accuracy of documentation or coding**	[If you looked back in the chart you may realize] “Oh. I treated that person, ‘cause they also had ‘*buh, buh, buh’* [e.g, other health issues]. By the time I wrote my report, I totally forgot about that. I just wanted to get it done, because it was 10 o’clock at night and I’d already seen 50 people.” (Provider #2) “…they [other clinicians] see a patient, they move on to the next patient quickly, so they don’t go back and double-check their codes. … I would say all of them need help with their coding.” (Provider #3) “…maybe we were 100 patients in, and we were 80 charts behind because it’s the top of flu season. We didn’t get to write our charts until the end of the day, and we forgot to put something in.” (Provider #7)
**Reviewing metric with clinicians allows identification of issues with diagnosis and coding**	“[A clinician] … kept putting in serous otitis media, instead of suppurative otitis media. I said, ‘Why are you doing this?’ She said, ‘Well, I went during my clinicals and shadowed ENT, that’s all they used.’ I’m like, ‘Yes, ‘cause they’re seeing chronic ear infections, which is serous, not suppurative. You were seeing a suppurative [infection].’ She just didn’t understand the difference between the diagnoses. Then I kinda guided them [to choose a different code].” (Provider #3) “I’m going to be honest in nurse practitioner school, we surely didn’t get a lot of coding education. And even through orientation, it was less than an hour. … The newer hires that are fresh from graduating and things like that, it’s very, very overwhelming for them.” (Provider #7) “To me, if I prescribed antibiotics for … [a viral illnesses], it would mean that I put the wrong diagnosis code for the patient.” (Provider #6)
**Feedback is valued by some clinicians but can also be overwhelming**	“… really, the more information you give is probably better. Just because sometimes, some of this stuff, depending on where we are in our cycle of what feedback we’re getting … millions of other things. The more attention you can get, I think it’s better. The more [data] you can put in, the easier you make it, and more direct, really.” (Provider #5) “I’ve always been a big fan of like, here’s the standard or what we’re looking for. I don’t mind getting help with the standard.” (Provider #2)

### Feedback is valued by some clinicians but can also be overwhelming

Some providers valued receiving metric feedback and described that the feedback made them conscious of what they were prescribing in relation to AS guidelines. For example, one NP (#7) noted “more feedback is more learning opportunities.” However, other providers noted that frequent feedback can also be overwhelming, given the many demands on their time and attention. Since feedback was delivered by email in a high-email-volume context, providers suggested communicating feedback in as simple and direct a way as possible and drawing attention to the critical point.

### Difficulty of interpreting metric based on concerns about accuracy of documentation or coding

Providers also reported some difficulty in interpreting the metric feedback or questioned the validity of the data. These concerns related to questions about the accuracy of documentation or coding in a busy clinical environment or when they needed to catch up on documentation for a number of charts.

### Metric may be prone to “gaming”

Some providers described shifting the ICD-10 codes they used in response to provider feedback, to perform better on the metric in the future. In the words of one PA, “I haven’t changed how I prescribe or treat. I’ve only changed how I play the computer game.” (#1) Thus, the “never-event” metric may be prone to ‘gaming’.

### Reviewing metric with clinicians allows identification of issues with diagnosis and coding

Finally, interviews allowed us to identify unexpected insights highlighting opportunities to support providers on both diagnosis and coding (eg, distinguishing serous from suppurative otitis media). Some providers also reported they appreciated learning when they were incorrectly coding, or felt their coding education had been limited. In contrast to the coding shift described above, several described that if providers were coding incorrectly, it identified a need for education regarding the diagnostic processes related to those codes.

## Discussion

In this quasi-experimental study across 7 walk-in clinics, implementation of a multifaceted intervention, which primarily consisted of provider feedback with peer-comparison, was associated with a temporary decrease in risk-adjusted antibiotic use for all visits and, over time, for never-event visits. We found that some clinicians appreciated the feedback from the “never-event” metric and discussed ways it might impact their prescribing decisions and reduce antibiotic use. However, both our quantitative and qualitative data suggest that some clinicians also changed their coding practices to avoid detection by the metric. Our study is novel in using mixed methods to evaluate the use and acceptability of a metric among walk-in clinic providers, including NPs and PAs. The literature demonstrates that provider feedback and peer-comparison can be an acceptable and effective intervention for changing clinical practice for antibiotic prescribing in outpatient settings.^14,15^ In our study, providers sometimes questioned the validity of the metric and the data underlying it. Similar skepticism has also been expressed in studies with outpatient primary care physicians and pediatricians.^16,17^ In both of these studies, participants also suggested that “gaming” could or did occasionally happen.

Our study similarly found that, in aggregate, providers shifted their diagnostic coding in response to receiving feedback with peer-comparisons. This finding aligned with our qualitative findings, which indicated that some providers changed their coding practices in response to receiving the feedback emails. While Madaras-Kelly et al also identified diagnostic shifting, others have not reported similar coding shifts.^7^ In general, coding habits have been shown to vary by provider and be related to diagnostic and prescribing practices.^18–20^

We speculate that diagnostic shifting occurred in our study because of the way in which our metric was implemented. First, our intervention did not include a robust education component to increase providers’ knowledge on how to prescribe antibiotics optimally and on how to communicate effectively with patients. In addition, providers’ performance on the metric was discussed in reviews with their supervisors, who provided feedback on diagnostic coding and antibiotic-prescribing. It is possible that the decline in unnecessary antibiotic prescribing and the changes in coding both were influenced by such discussions. The changes we observed in coding may reflect improved accuracy in coding (ie, more frequently choosing the correct diagnosis) as well as purposely avoiding certain diagnostic codes to prevent being flagged for feedback. This suggests that provider feedback and peer-comparison may need to be monitored for changes in both prescribing and coding.

This study has several limitations. First, because this study was a quasi-experimental trial, we cannot conclude causality. Since engagement with the dashboard could not be measured, we cannot associate engagement with impact. It is possible that other temporal trends, such as community outbreaks of specific respiratory pathogens, could have affected clinicians’ practices. Second, the intervention period started before the COVID-19 pandemic ended, and it is unclear how pandemic-related changes in care delivery affected the practices we measured. Third, all data was collected in clinics affiliated with one institution and thus may not be generalizable to all walk-in clinics; similarly, qualitative sample bias likely exists in that providers who were more interested in stewardship may have been more motivated to participate. Fourth, we used data from the electronic medical record, which included ICD-10 codes applied to clinic visits, and potentially diagnoses were mis-coded. We could not measure patient symptoms that might have affected antibiotic prescribing. Fifth, turnover is frequent, so not all providers may have received the same education regarding the ‘never-event’ prescribing metric.

In conclusion, delivering feedback to walk-in clinic providers on their antibiotic use was associated with temporary reductions in antibiotic-prescribing across all visits but also changes in diagnostic coding (ie, “gaming”). Antibiotic stewardship programs should monitor for changes in both when implementing new outpatient metrics. Future studies should investigate barriers to clinicians’ accurate use of diagnostic coding and methods to optimize provider feedback to encourage practice change.

## Supporting information

10.1017/ash.2026.10365.sm001 Percival et al. supplementary material 1 Percival et al. supplementary material

10.1017/ash.2026.10365.sm002 Percival et al. supplementary material 2 Percival et al. supplementary material

## Supplementary material

The supplementary material for this article can be found at https://doi.org/10.1017/ash.2026.10365.

## Research transparency and reproducibility

The corresponding author will share data used for this study on reasonable request.
