# Supplementary material for: Mixed-method evaluation of interactive provider dashboards for comparison of outpatient antibiotic prescribing for respiratory and otic conditions in walk-in clinics
Source: Antimicrob Steward Healthc Epidemiol. 2026 May 11;6(1):e128. doi: 10.1017/ash.2026.10365 (PMC13162070; doi:10.1017/ash.2026.10365)
Supplement: Percival et al. supplementary material 2 — Percival et al. supplementary material [file S2732494X26103659sup002.docx]

Supplementary Figure 1. Never antimicrobial event prescription rate peer-comparison


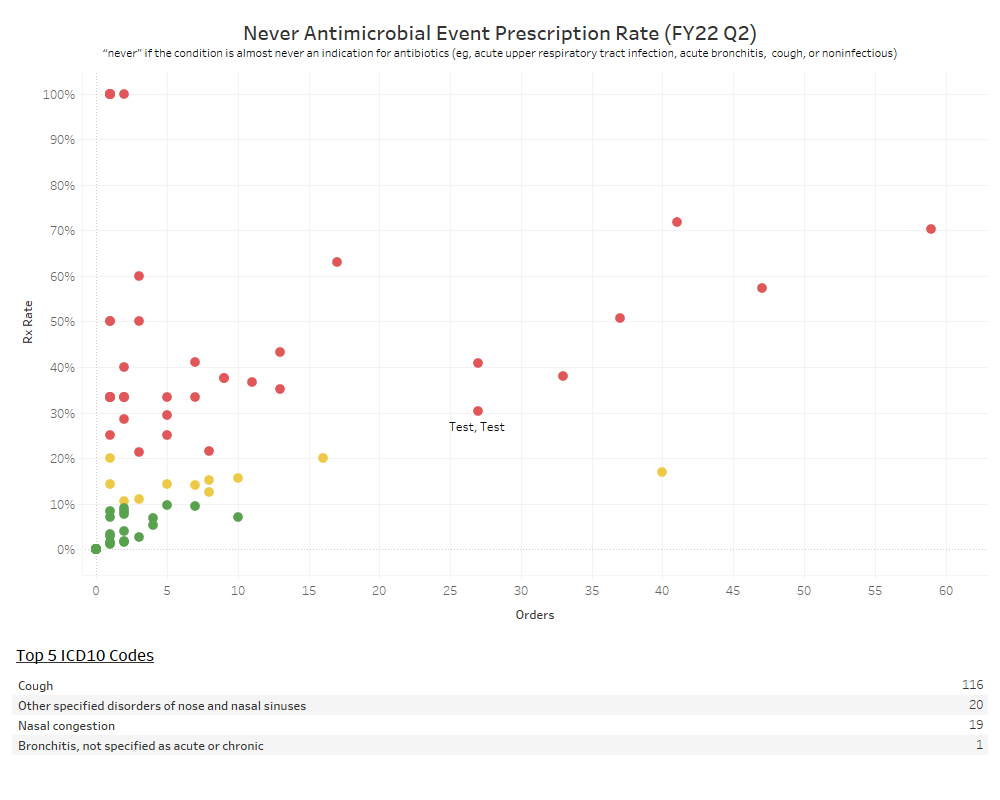
 The X-axis is the number of antibiotic orders written during the quarter for a never-event condition. The Y-axis the Prescription (Rx) rate which is calculated as the # of antibiotic orders for a never-event divided by number of encounters for a never-event during the quarter.

Top ICD10 codes represents the top never-event ICD10s used on antibiotic prescriptions during the quarter for all UC/QC clinics. Will display up to top 5 codes

Test, Test is a fictious name to demonstrate how it would look to an individual provider. Their dot would be labeled with their name while all other dots are anonymous.

Green indicates a ≤10% prescribing rate of antibiotics for never-event conditions

Yellow indicates a 10.1 to 20% prescribing rate of antibiotics for never-event conditions

Red indicates a >20% prescribing rate of antibiotics for never-event conditions

Supplementary Figure 2. Never antimicrobial event clinic prescription rate trend


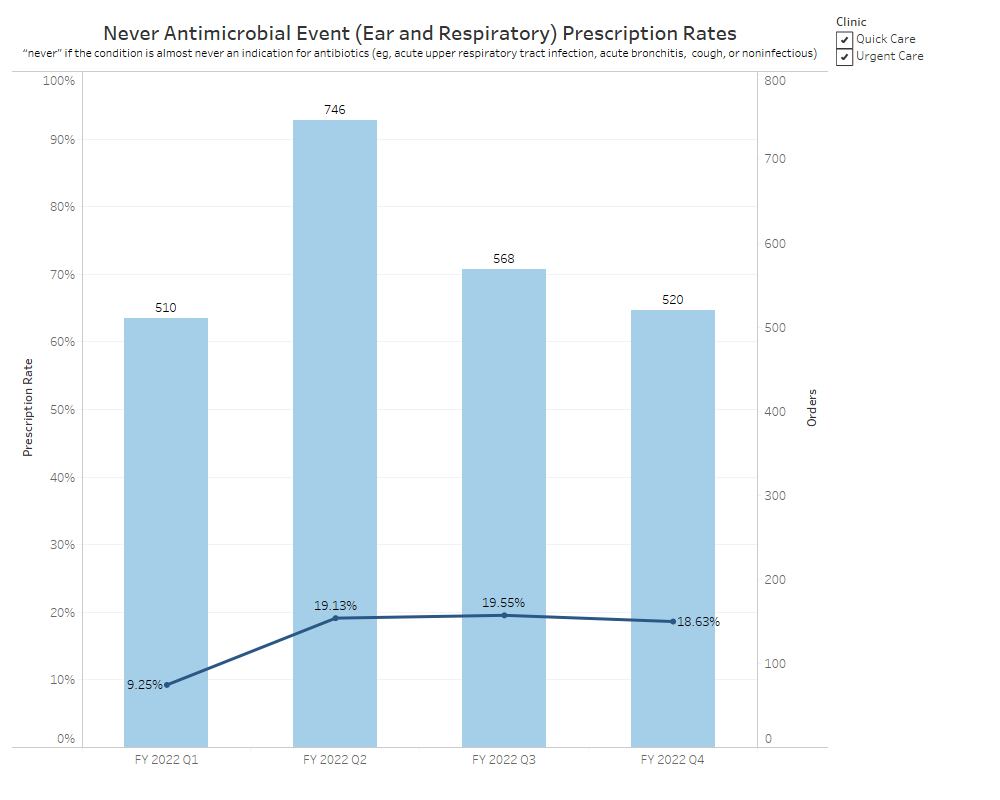


Bars are the number of antibiotic orders in a month for never-events in all UC/QC clinics.

Line is rate of prescriptions for never-events all UC/QC.

Supplementary Figure 3. Never antimicrobial event provider and clinic prescription rate trend


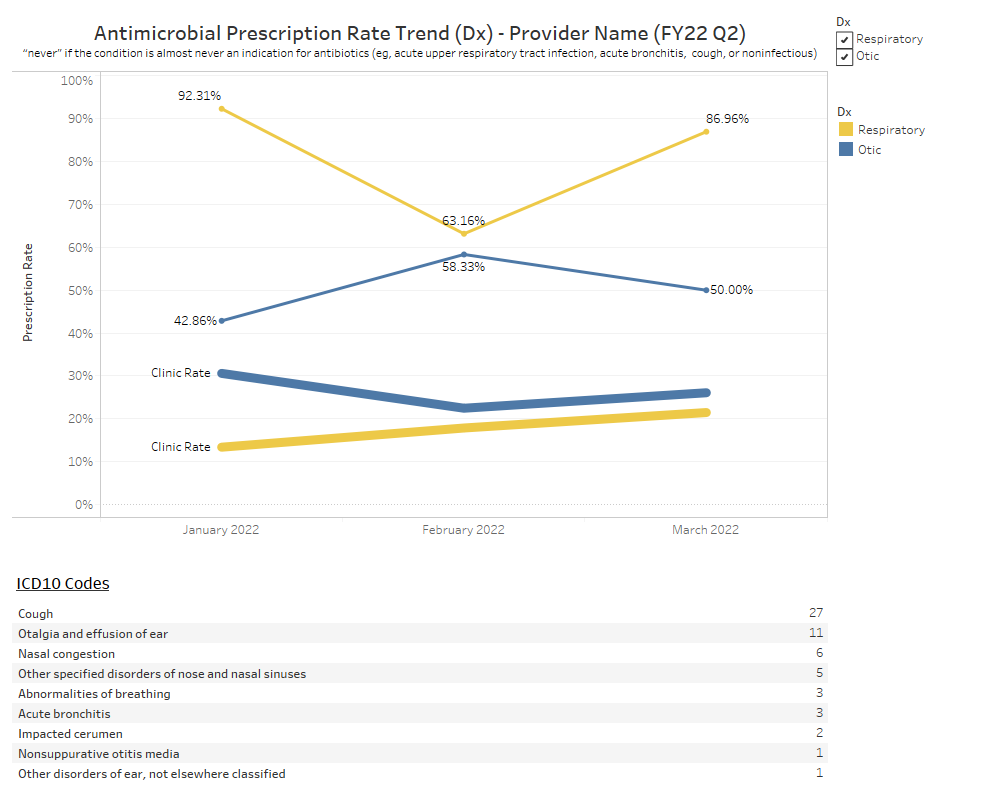


Narrow line represents provider specific rate; Bold line represents all Urgent and QuickCare clinics rate. Top ICD10 codes are the provider specific top ICD10 codes for never-events utilized during the quarter. This was specific to the provider when they logged into Tableau^®^, their name would appear where this example shows provider name.


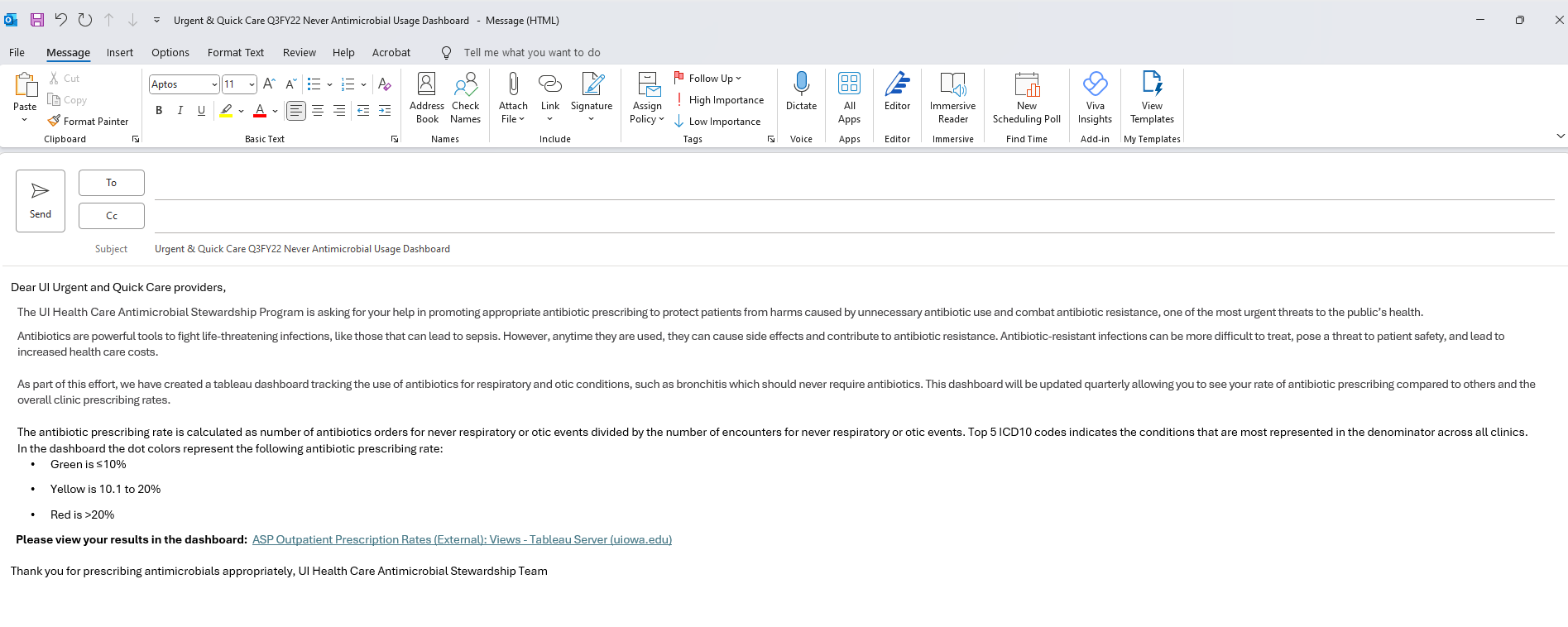
Supplementary Figure 4. Quarterly feedback email

Supplementary Table 1. Antibiotics prescribed by Urgent Care and QuickCare clinicians that were captured

| Amoxicillin |
| --- |
| Amoxicillin-clavulanate |
| Ampicillin |
| Azithromycin |
| Cefaclor |
| Cefadroxil |
| Cefdinir |
| Cefixime |
| Cefpodoxime |
| Cefprozil |
| Ceftriaxone given as an intramuscular injection |
| Cefuroxime |
| Cephalexin |
| Ciprofloxacin |
| Clarithromycin |
| Clindamycin |
| Dicloxacillin |
| Doxycycline |
| Erythromycin |
| Gemifloxacin |
| Levofloxacin |
| Linezolid |
| Minocycline |
| Moxifloxacin |
| Ofloxacin |
| Penicillin G benzathine as an intramuscular injection |
| Penicillin V potassium |
| Sulfamethoxazole/trimethoprim |
| Tetracycline |

Oral antibiotics not included: fidaxomicin, fosfomycin, metronidazole, nitrofurantoin, rifampin, vancomycin, any antifungals, antiparasitic agents or any antivirals.

Supplementary Table 2: Types and frequency of antibiotics prescribed across 7 walk-in clinics, 2018-2023*

| **Type of antibiotic** | **Number of prescriptions written** |
| --- | --- |
| Penicillins, including amoxicillin, dicloxacillin, and penicillin | 31,984 |
| Amoxicillin-clavulanate | 25,339 |
| Cephalosporins | 24,024 |
| Tetracyclines | 12,452 |
| Trimethoprim-sulfamethoxazole | 5,864 |
| Fluoroquinolones | 3,435 |
| Macrolides | 1,660 |
| Clindamycin | 799 |
| Other | 11 |

*Patients could be prescribed more than one antibiotic type during a single visit.

Supplementary Figure 5. Model-adjusted trends in coding for never-events frequency overlaid on observed coding for never-events across 7 walk-in clinics, 2018-2023


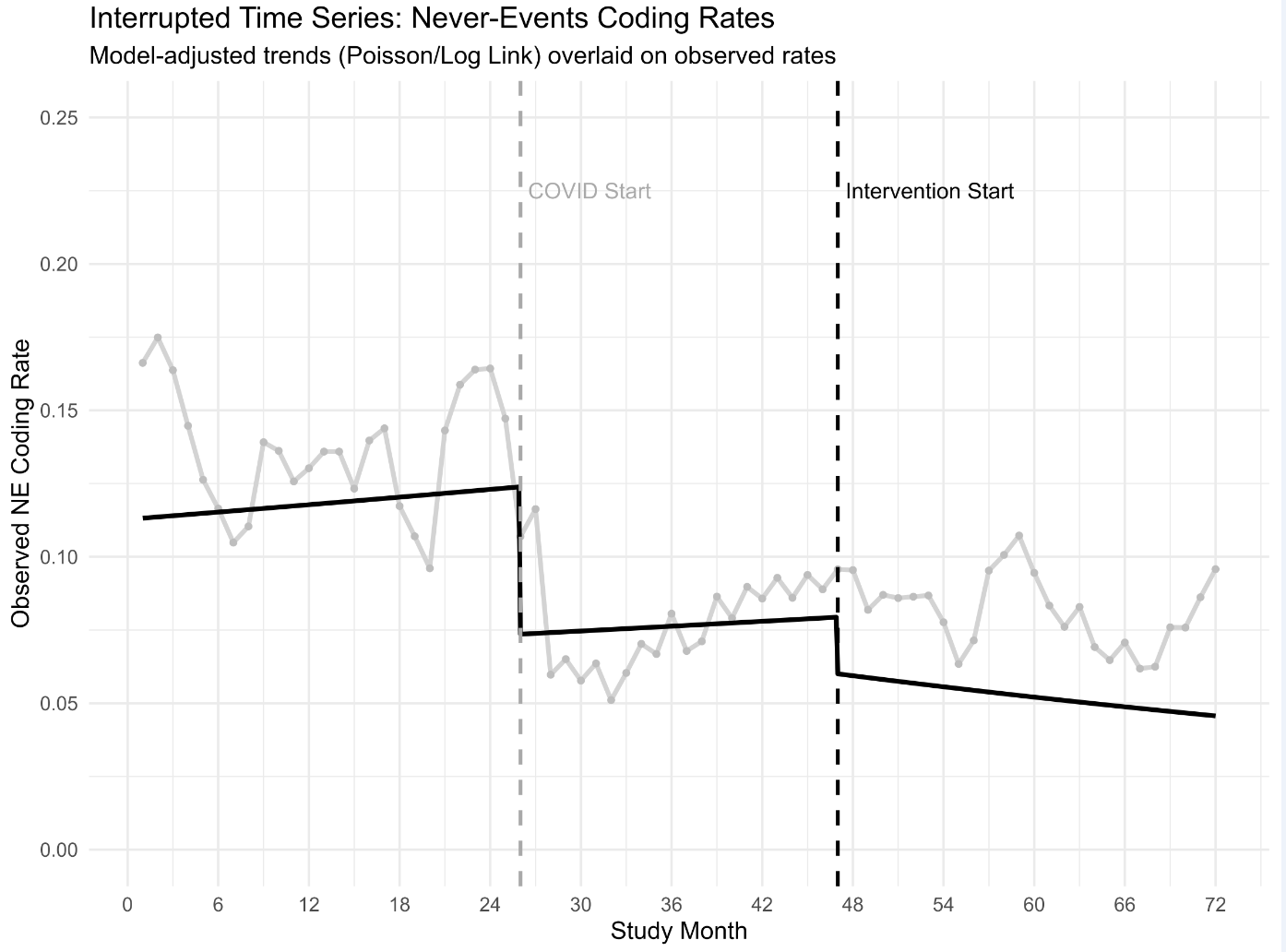


Monthly observed never-event (NE) coding rate, from walk-in clinics during baseline months 1-25 (January 2018-January 2020), COVID-19 starts month 26 (February 2020), Intervention months 47-72 (November 2021-December 2023)

Supplementary Figure 6. Model-adjusted trends in antibiotic-prescribing frequency for never-events overlaid on observed prescribing rates for never-events across 7 walk-in clinics, 2018-2023


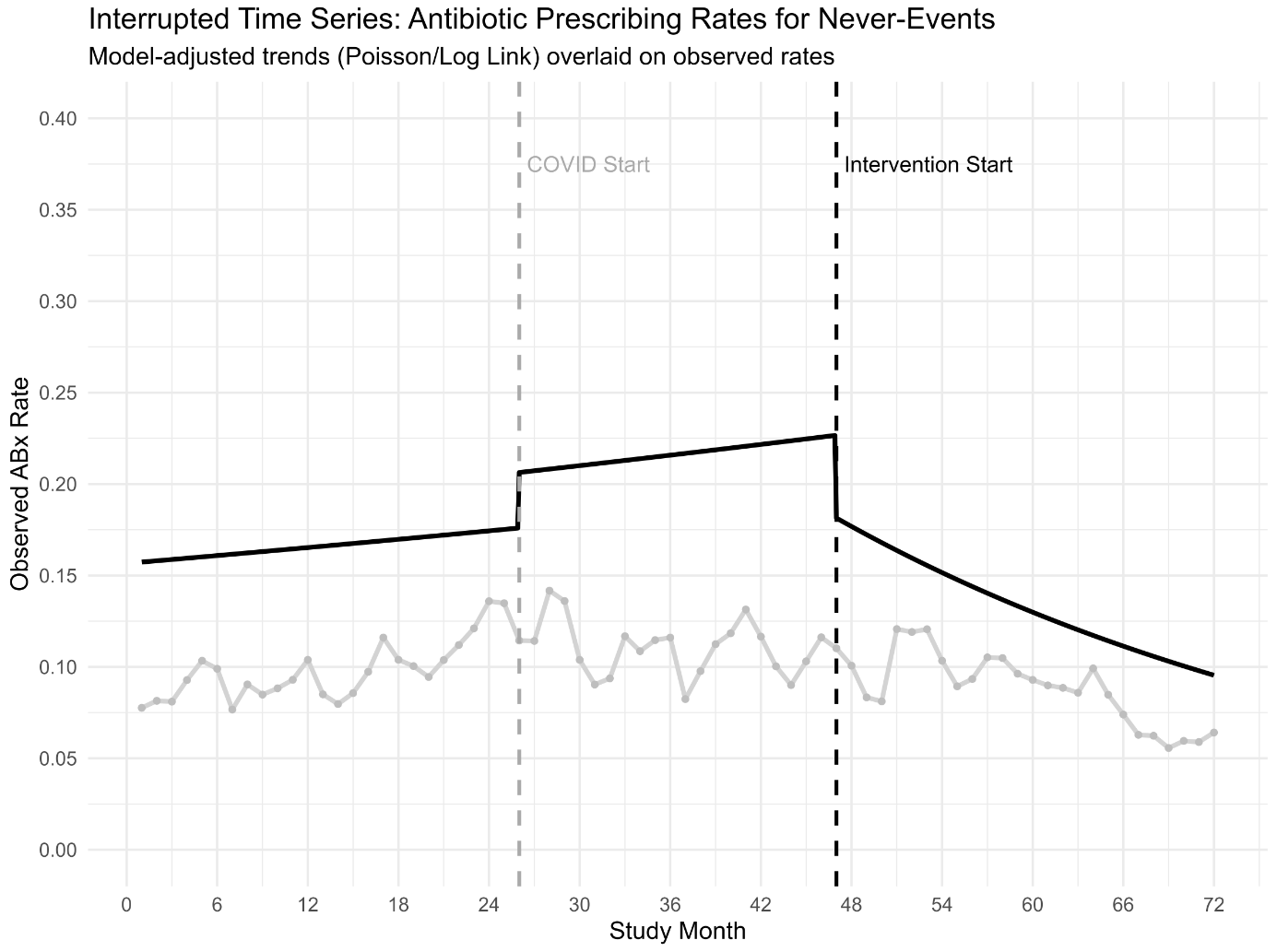


Monthly observed antibiotic prescribing rate (ABx rate) for an antibiotic that could be used for a respiratory infection for never-events, from walk-in clinics during baseline months 1-25 (January 2018-January 2020), COVID-19 starts month 26 (February 2020), Intervention months 47-72 (November 2021-December 2023)
